# Common millet and soybean intercropping with bio-fertilizer as sustainable practice for managing grain yield and quality

**DOI:** 10.3389/fnut.2023.1267928

**Published:** 2023-11-29

**Authors:** Milena Šenk, Milena Simić, Dušanka Milojković-Opsenica, Milan Brankov, Miodrag Tolimir, Igor Kodranov, Vesna Dragičević

**Affiliations:** ^1^R&D Department, Group for Agro-Ecology and Cropping Practices, Maize Research Institute “Zemun Polje”, Belgrade, Serbia; ^2^Department of Analytical Chemistry, Faculty of Chemistry, University of Belgrade, Belgrade, Serbia

**Keywords:** grain, sowing pattern, crop combinations, land equivalent ratio, elements, antioxidants, anti-nutrients, bio-availability

## Abstract

Climate changes are one of the biggest threats to food security. Sustainable agriculture, focused on eco-friendly practices for highly efficient food production, enables greater resilience and safety. This study experimented on intercropping and bio-fertilizer application as convenient ecological solutions for crop yield stability and quality. The experiment was conducted during 2018 and 2020 with soybean and common millet sown in three sowing patterns: alternating rows, alternating strips 1 (2 rows of soybean + 2 rows of millet), and alternating strips 2 (2 rows of soybean + 4 rows of millet), as well as sole crops (control), with or without a bio-fertilizer Coveron. Grain yield and nutrient grain yield response were calculated through land equivalent ratio (LER) and element-LER (E-LER), while quality was estimated based on the concentration of antioxidants (phytate phosphorus, total phenolic compounds, and yellow pigment) and elements in grains, including potential bio-availability of essential elements. Results revealed LER values to be >1 for all sowing patterns, with the highest one achieved in alternating strips 1 (1.38) together with a greater level of all antioxidants in millet grain. Intercropping significantly enhanced Fe and Mn accumulation in both crops and simultaneously decreased the concentration of potentially toxic elements (Al, Cr) in millet grain. Potential bio-availability of essential elements, expressed through the ratio between phytic acid and Ca, Mg, Fe, and Zn revealed smaller values in intercropped soybean and millet with the bio-fertilizer. The bio-fertilizer also increased the concentration of some micro-elements in millet grain, classifying it as a highly dependent plant to microbial inoculation. Interaction of intercropping and bio-fertilizer was most pronounced for LER, E-LER, and accumulation of Fe and Mn in grains. These results highlighted the benefits of soybean–common millet intercropping, especially in combination with the bio-fertilizer, in light of enhanced land utilization and nutrient absorption, thus increasing the resilience of soybean and millet under dry land conditions and low-input systems toward stability and food security.

## 1 Introduction

Chemical inputs, as a common part of industrial agriculture, have reached a tremendous scale, polluting the environment and threatening human health at the same time. Consequently, much attention worldwide is focused on sustainable agriculture without compromising ecological resources, supporting agro-ecosystem services for highly efficient food production (1). Intercropping and use of bio-fertilizers represent eco-friendly practices that promote sustainability since both have an important role in improving soil fertility, nutrient use efficiency, soil structure, and microbial diversity, thereby improving soil health (2, 3). Furthermore, intercropping and bio-fertilizers enhance crop productivity and quality (4, 5), while their combination has the potential to be used as an eco-friendly way of establishing harmonization between the environment and reasonable yields (6).

Intercropping implies growing two or more crops simultaneously in the same field. Combining legumes and cereals in intercropping has been successfully used due to dissimilar growing patterns and nutrient requirements, particularly when nitrogen was considered. The major advantages of this combination reflect through yield improvement and stability, together with enhanced light, water, and nutrient utilization, improved soil fertility, as well as weed and pest control (7, 8).

Bio-fertilizers include microorganisms capable of enhancing the availability of nutrients to the plant (9). Arbuscular mycorrhizal fungi (AMF), plant growth-promoting rhizobacteria (PGPR), and *Trichoderma* sp. are recognized as beneficial soil microorganisms which express favorable effects on host plants (10). They might promote plant growth, nutrient uptake, and stress resilience through different mechanisms (synthesizing secondary metabolites, producing plant hormones, mobilizing nutrients, increasing the root area with colonization, etc.). Therefore, bio-fertilizer use can improve crop fitness while simultaneously reducing environmental footprint (10, 11).

Soybean [*Glycine max* (L.) Merr.] and common millet (*Panicum miliaceum* L.), commonly known as proso millet, hog millet, or broomcorn millet, are two important crops with valuable nutritive traits. Soybean is an excellent protein source, while millet stands out among cereals in regard to protein content. Both of them are rich in minerals: phosphorus, magnesium, iron, zinc, boron, manganese, copper, etc. (12, 13). Since soybean and millet are also recognized as a source of anti-nutrients, such as phytic acid and phenolic compounds, their nutritional value should be improved (14, 15). Increasing the mineral content in grain is just one step in the way to boosting food quality, while reducing the concentration of anti-nutrients, which negatively affects the bio-availability of minerals from the intestine of monogastric organisms, including humans, is an important step too (16). Noting that both phenolic compounds and phytic acid exhibit antioxidant properties and play an important role in the prevention of various diseases, optimal balance in their concentration is of the utmost importance (17). Reversely, yellow pigment (mainly consisting of carotenoids) (18) is desirable and should be fortified in grains due to pronounced antioxidant activity and beneficial effect on mineral bio-availability, thus contributing to the mitigation of micronutrient malnutrition (19, 20). The ability of soybeans to fix the atmospheric nitrogen contributes to improved soil fertility (21), and therefore, it could be combined with non-leguminous crops, particularly when external inputs are limited, fostering sustainability (7). On the other hand, common millet has high ecological resilience and great potential for growing in dry conditions, contributing to global food security (22). Therefore, soybean and millet could be successfully intercropped.

The soybean and common millet combination in intercropping has been rarely investigated so far and is relatively new, particularly when grain production for human consumption was considered. The results of some studies showed a positive effect on land utilization (23, 24) and grain quality (25). Consequently, the goal of this research was (1) to get information about the complementarity of these two crops; (2) to examine the integrated effect of intercropping and bio-fertilizer on grain productivity, i.e., yield and quality; and (3) to point out the most promising combination of intercropped soybean and common millet, together with the bio-fertilizer, as a way to boost grain yield and quality in the low-input system, supporting sustainability. The focus of this research was grain yield and quality estimated through land equivalent ratio (LER), status of nutrients and anti-nutrients (elements, yellow pigment, total phenolic compounds, and phytate phosphorus), elements LER (E-LER), and assessment of potential bio-availability of essential elements (Ca, Mg, Fe, and Zn).

## 2 Materials and methods

### 2.1 Experimental site and soil properties

The experiment was set up at the experimental field of the Maize Research Institute “Zemun Polje,” Belgrade vicinity (44°52′ N; 20°20′ E), Serbia, during 2018 and 2020. The soil was slightly calcareous chernozem with 30% silt, 17% clay, and 53% sand. Properties of the 0–30 cm soil layer were determined before sowing and showed 4.32% organic matter (26), pH 7.3, and 1.38% total CaCO_3_ (27). Information about the concentration of available N and P and extractable K, Ca, Mg, S, B, Al, Cr, Mn, Fe, Co, Ni, Cu, Zn, and Se, for 2018 and 2020, are given in Table 1. N was determined by the method of Scharpf and Wehrmann (28), P according to Watanabe and Olsen (29), while the content of other available elements was analyzed on ICP-OES (Thermo Scientific iCAP 6500 Duo, Thermo Fisher Scientific, USA) using Mehlich 3 solution for extraction (30).

**Table 1:** Concentrations of available N and P (kg ha^−1^), as well as extractable macro- and micro-elements (mg kg^−1^), in soil for 2018 and 2020, at the Zemun Polje experimental field, before sowing.

**Year**	**N**	**P**	**K**	**Ca**	**Mg**	**S**	**B**	**Al**	**Cr**	**Mn**	**Fe**	**Co**	**Ni**	**Cu**	**Zn**	**Se**
2018	71.9	32.7	258.1	4,002	299.5	72.4	14.1	263.0	0.17	185.4	63.2	1.5	2.2	4.7	4.3	0.03
2020	117.9	41.2	359.7	3,856	338.7	70.3	11.1	257.1	0.33	207.6	37.1	1.7	4.3	4.4	2.9	0.08

### 2.2 Experimental design

Soybean, *var*. Selena (Maize Research Institute “Zemun Polje,” Serbia), and common millet, *var*. Biserka (Institute of Field and Vegetable Crops, Novi Sad, Serbia), were used in the experiment. The trial was set up as a completely randomized block design (RCBD) with four replications. Elementary plots were encompassed 3 × 5 m, except AS2, which was set up as 4 × 5 m (due to the sowing pattern). Sowing was performed on 3rd May 2018 and 22nd April 2020. The experiment from 2019 was excluded due to the heavy rain period and flood during the first week of May, which obstructed proper germination. Experimental design, including combinations, sowing pattern (SP), sowing density, and inter-row distance, is presented in Table 2.

**Table 2:** Experimental design.

**Experimental combination /Sowing pattern**		**Crop combination**		**Sowing density (plants ha** ^**−1**^ **)**		**Inter-row distance (cm)**		
		**Soybean**	**Millet**	**Soybean**	**Millet**	**Soybean–soybean**	**Soybean–millet**	**Millet–millet**
S1	S1 + BF	Sole crop		440.000		50		
S2	S2 + BF		Sole crop		2,640.000			25
AR	AR + BF	Alternating rows		220.000	660.000		50	
		One row	One row					
AS1	AS1 + BF	Alternating strips		352.000	1,056.000	50	25	25
		Two rows	Two rows					
AS2	AS2 + BF	Alternating strips		195.556	1,173.333	50	50	25
		Two rows	Four rows					

S1, sole crop of soybean; S2, sole crop of millet; AR, alternating rows of soybean and millet; AS1, alternating strips of two rows of soybean and two rows of millet; AS2, alternating strips of two rows of soybean and four rows of millet; BF, bio-fertilizer.

The influence of bio-fertilizer Coveron (BF), Hello Nature International Srl, Italy, containing microorganisms species *Glomus* sp., *Trichoderma atroviride*, and plant growth-promoting rhizobacteria, was also included in the trial. The bio-fertilizer was dissolved in water (50 g in 800 mL) and solution was applied per 100 kg of seeds, before sowing. BF was applied in all intercrop variants and in all four replications, in addition to the same plots without BF (Table 2).

The experiment was conducted in dry land conditions, without fertilization or application of any other agro-chemical. Weeds were removed by hoeing, as needed (2–3 times during vegetation).

### 2.3 Harvesting and data collection

Harvesting of half area per replication was performed manually at the full maturity of crops (beginning of August 2018 and the end of July 2020 for common millet, and mid of October in both years for soybean). The grain yield (GY, t ha^−1^) was measured for each plot and calculated to 13% moisture content. LER was calculated according to the formula given by Mead and Willey (31):


$$\begin{array}{l} LER=\frac{Y_{A}}{S_{A}}+\frac{Y_{B}}{S_{B}} \end{array}$$

Where Y_A_ and Y_B_ represent individual crop yields in intercropping, while S_A_ and S_B_ indicate sole crops yields.

### 2.4 Chemical analysis

Grain samples were milled on Perten 120—Sweden (particle size < 500 μm). All spectrophotometrical analyses were performed in four replications. Concerning preparation for phytate phosphorus (Pphy) and total phenolic compounds (TPC) determination, soybean flour was first defatted by extraction with petroleum ether for 14 h. Then, 0.25 g of sample was extracted with 10 ml 5% TCA, within 1 h, and centrifuged for 15 min at 12,000 rpm and 4°C (Dynamica Velocity 18R, Versatile Centrifuge, Australasia, Dynamica, Pty Ltd, Australia). The Pphy concentration was determined using Wade reagent (0.3 g FeCl_3_ × 6 H_2_O + 3 g 5′sulfosalycilic acid L^−1^) at λ = 500 nm, on Biochrom Libra S22 UV/Vis Spectrophotometer (Biochrom, UK), (32), and results were expressed as mg g^−1^ of dry matter (DM). Dilution of prepared extract for reaction was 1:5 for millet solution and 1:16 for soybean solution. TPC was determined from the same extract which was used for Pphy determination, in a reaction with 100 μL of 0.008 M K_3_Fe(CN)_6_ and 100 μL of 0.05 M FeCl_3_ in 0.1 M HCl, at λ = 720 nm. Dilution for millet solution was 1:30, while for soybean solution 1:20. Results were expressed as μg of 3-hydroxy-4-methoxycinnamic acid per g of DM (33). The extraction of yellow pigment (YP) was performed with saturated 1-butanol for 0.5 h and, after centrifugation at 10,000 rpm and 4°C for 10 min, absorbance was read at λ = 436 nm. Results were expressed as μg of β-carotene equivalent (βCE) per g of DM (34).

Elemental assessment was accomplished in triplicate: 0.5 g of milled sample was dissolved in 5 ml conc. HClO_4_ + 5 ml conc. HNO_3_, and left overnight in the dark. Then, wet digestion was performed on the Behrotest K16 digestion unit (behr Labor-Tecnik GmbH, Düsseldorf, Germany) by heating for 4 h (it started at 50°C, and every 0.5 h, the temperature was gradually raised for an additional 50°C until 250°C was reached, after which the temperature was maintained) until samples become clear. After cooling, the extract was diluted with ultra-clean water (1:36) and prepared for inductively coupled plasma (ICP) analysis. The concentration of macro-elements (Ca, Mg, and S) was determined by inductively coupled plasma optical emission spectrometry (ICP-OES) using a Thermo Scientific iCAP 6500 Duo (Thermo Fisher Scientific, USA), while micro- and trace elements (B, Al, Cr, Mn, Fe, Co, Ni, Cu, Zn, Se, Mo, Cd, and Pb) were determined using inductively coupled plasma mass spectrometry (ICP-MS), iCap Q, Thermo Scientific, UK. ICP instrumental conditions are shown in Table 3. A multi-element stock solution (Major Elements Stock, EPA Method Standard, VHG Labs) and stock solution for S (6020A ICS Stock, EPA Method Standard, VHG Labs) were used to prepare standard solutions for ICP-OES. Standard solutions for ICP-MS were made from Multi-Element Plasma Standard Solution 4 (Alfa Aesar), Selenium Standard for AAS (Fluka), and Molybdenum Plasma Standard Solution (Alfa Aesar).

**Table 3:** ICP instrumental conditions.

**ICP-OES**			**ICP-MS**	
**Parameter**		**Setting**	**Parameter**	**Setting**
Pump winding		Orange/white tygon sample White/white tygon sample	Spray chamber temperature	2.7°C
Pump rate		50 rpm	Peristaltic pump speed	40 rpm
Nebulizer		Standard concentric	Cool flow	14 Lmin^−1^
Nebulizer argon pressure		0.6 L min^−1^	Plasma power	1,550 V
Spray chamber		Standard cyclonic	Auxilliary flow	0.8 Lmin^−1^
Center tube		2.0 mm	Nebulizer Flow	1 Lmin^−1^
Torch orientation		Duo	Torch horizontal Position	0.47
RF forward power		1,150 W	Torch vertical Position	−0.07
Purge gas		Argon	Extraction Lens 2 (V)	−89 V
Coolant flow		12 L min^−1^	CCT focus lens	−3
Auxiliary flow		0.5 L min^−1^		
Integration times	High wavelengths	5 sec		
	Low wavelengths	15 sec		
Analysis mode		Speed		

E-LER, as an indicator of the effect of intercropping on elements yield per land area (35), was calculated for examined elements, except for Cd and Pb, whose concentrations were below the detection limit and not considered for further statistical analysis.

### 2.5 Statistical analysis

The obtained results were processed using analysis of variance (ANOVA). Means were tested by Fisher's least significant difference (LSD) at the significance level of *p* < 0.05. The molar ratios between phytic acid (PA) and essential elements (PA/Mg, PA/Ca, PA/Fe, and PA/Zn) were presented as a mean ± standard deviation (SD). Principal component analysis (PCA) was used to assess the interdependence among intercrop combinations and BF regarding grain composition, i.e., concentrations of Pphy, TPC, YP, and examined elements, using SPSS 15.0 (SPSS Inc., Chicago, IL) for Windows (36).

### 2.6 Meteorological conditions

Meteorological conditions during the growing seasons pointed to a higher average temperature in 2018 than in 2020 and a 10-year average (for 1.4 and 1.5°C, respectively) (Table 4). May 2018 and April 2020 were quite drier compared to the 2008–2017 average. However, higher precipitation was present in June in both years. Higher precipitation level was present in July 2018 and August 2020, while September in both years was drier with higher average temperature, compared to the 10-year average.

**Table 4:** Mean temperature and precipitation amount at Zemun Polje during the growing seasons of 2018 and 2020, and the 2008–2017 average.

**Months**	**Average temperature (** **°** **C)**			**Precipitation sum (mm)**		
	**2018**	**2020**	**2008–2017**	**2018**	**2020**	**2008–2017**
April	18.0	14.4	14.2	24.6	4.7	37.4
May	21.7	16.9	18.5	39.0	79.9	76.7
June	22.7	21.3	22.5	150.1	125.9	68.4
July	23.6	23.3	24.6	61.9	34.8	51.8
August	25.7	25.2	24.4	44.0	66.3	38.2
September	19.8	21.9	19.3	16.9	16.1	47.7
October	15.9	15.0	13.4	20.8	73.4	48.8
Aver./sum	21.1	19.7	19.6	357.3	401.1	369.0

## 3 Results

### 3.1 Yield parameters

The intercropping expressed a significant impact on the GY of both crops (Table 5). The greatest GY of soybean was obtained in AS1 (6.68 t ha^−1^), while the lowest one was in S1. BF, year (Y), and their interaction did not express a significant impact on soybean GY.

**Table 5:** Analysis of variance for the effects of sowing pattern (SP), bio-fertilizer (BF), year (Y), and their interaction on the variation of grain yield (GY), land equivalent ratio (LER), concentration of phytate phosphorus (Pphy), total phenolic compounds (TPC), and yellow pigment (YP) in soybean and common millet grains.

	**Soybean**				**Common millet**				
**Sources of variation**	**GY**	**Pphy**	**TPC**	**YP**	**GY**	**Pphy**	**TPC**	**YP**	**LER**
	**(t ha** ^−1^ **)**	**(mg g** ^−1^ **)**	**(**μ**g g**^−1^**)**	**(**μ**g g**^−1^**)**	**(t ha** ^−1^ **)**	**(mg g** ^−1^ **)**	**(**μ**g g**^−1^**)**	**(**μ**g g**^−1^**)**	
S1/S2	4.22^a^	4.08^ns^	459.10^ns^	11.37^ns^	2.91^b^	2.48^a^	48.22^a^	8.01^b^	
AR	5.09^b^	3.94^ns^	492.80^ns^	9.51^ns^	1.86^a^	2.64^a, b^	81.20^b^	7.15^a^	1.03^a^
AS1	6.68^c^	3.82^ns^	525.50^ns^	11.76^ns^	2.31^a, b^	2.71^b^	52.34^a^	8.09^b^	1.38^b^
AS2	5.32^b^	3.88^ns^	547.80^ns^	10.47^ns^	2.41^a, b^	2.58^a, b^	43.35^a^	6.63^a^	1.06^a^
BF	5.04^ns^	4.04^b^	485.50^ns^	10.78^ns^	2.44^ns^	2.58^ns^	61.15^ns^	7.47^ns^	1.21^ns^
BF⊖	5.61^ns^	3.83^a^	527.10^ns^	10.78^ns^	2.31^ns^	2.62^ns^	51.41^ns^	7.47^ns^	1.10^ns^
2018	5.44^ns^	3.57^a^	283.11^a^	7.48^a^	2.86^b^	2.45^a^	55.03^ns^	7.17^a^	1.14^ns^
2020	5.22^ns^	4.29^b^	729.48^b^	14.07^b^	1.88^a^	2.75^b^	57.53^ns^	7.77^b^	1.18^ns^
***p*** **-value**									
SP	0.000	0.353	0.740	0.615	0.002	0.012	0.000	0.000	0.000
BF	0.063	0.049	0.485	0.999	0.537	0.355	0.062	0.981	0.151
Y	0.474	0.000	0.000	0.000	0.000	0.000	0.637	0.032	0.584
SP × BF	0.000	0.393	0.974	0.973	0.027	0.088	0.000	0.001	0.000
SP × Y	0.000	0.000	0.000	0.000	0.000	0.000	0.000	0.000	0.000
BF × Y	0.176	0.000	0.000	0.000	0.000	0.000	0.231	0.212	0.507
SP × BF × Y	0.000	0.000	0.000	0.000	0.000	0.000	0.000	0.000	0.002

S1, sole crop of soybean; S2, sole crop of millet; AR, alternating rows of soybean and millet; AS1, alternating strips of two rows of soybean and two rows of millet; AS2, alternating strips of two rows of soybean and four rows of millet; BF, bio-fertilizer; BF⊖, without bio-fertilizer. Different letters indicate significant differences at *p* < 0.05, ns, not significant.

Regarding millet, the highest GY was in S2 (2.91 t ha^−1^), while the lowest value was achieved in AR. Y significantly increased GY by 1 t ha^−1^, in favor of 2018. All interactions, SP × BF, SP × Y, BF × Y, and SP × BF × Y, influenced significant variability in millet GY.

For all intercrop combinations, LER values were ≥1 and revealed significant and the highest LER in AS1 combination (38% greater compared to the sole crops). Interactions SP × BF, SP × Y, and SP × BF × Y were significant for LER variations.

### 3.2 Concentration of phytate phosphorus, total phenolic compounds, and yellow pigment

For soybean, BF significantly increased only Pphy concentration for 0.21 mg g^−1^ (Table 5). However, Pphy, TPC, and YP were considerably affected by the year, pointing to higher values in 2020 than in 2018 for 0.72 mg g^−1^, 446.37, and 6.59 μg g^−1^, respectively. The significant impact of interactions SP × Y, BF × Y, and SP × BF × Y was expressed, too.

The concentrations of Pphy and TPC in millet grain were generally increased by intercropping, having the highest value in AS1 (2.71 mg g^−1^) and AR (81.20 μg g^−1^), respectively. YP was the greatest in the AS1 combination (8.09 μg g^−1^). However, Y affected Pphy and YP values, pointing to higher average concentrations in 2020 than in 2018 for 0.30 mg g^−1^ and 0.60 μg g^−1^, respectively. Concerning interactions, SP × BF had significant impact on TPC and YP concentrations, while significant variability in Pphy was influenced by BF × Y. Influences of SP × Y and SP × BF × Y interactions were significant for all examined parameters.

### 3.3 Elemental composition

According to the results obtained for soybean (Table 6), a significant SP effect was noticed for Mn and Fe concentrations, having the highest values of 32.47 mg kg^−1^ in AS1 and 92.83 mg kg^−1^ in AS2, respectively. The significant BF influence was expressed through higher Al and Co values, as well as lower S and Cr values (in BF). Y was significant for the accumulation of all examined elements, except for Mn and Se. On average, a greater concentration of macro- and micro-elements was in 2018 than in 2020 (except for Cr and Co, with significantly higher values in 2020). Interaction of SP × BF was significant only for the concentrations of Mn, Fe, and Co, while SP × Y and BF × Y induced variability of majority of examined elements. Interaction SP × BF × Y was significant for all elements.

**Table 6:** Analysis of variance for the effects of sowing pattern (SP), bio-fertilizer (BF), year (Y), and their interaction on the variation of macro- and micro-elements in soybean grain.

**Sources of variation**	**Ca**	**Mg**	**S**	**B**	**Al**	**Cr**	**Mn**	**Fe**	**Co**	**Ni**	**Cu**	**Zn**	**Se**	**Mo**
	**(g kg** ^−1^ **)**			**(mg kg** ^−1^ **)**										
S1	2.74^ns^	3.14^ns^	3.24^ns^	36.54^ns^	5.82^ns^	0.21^ns^	27.98^a^	81.04^a^	0.16^ns^	5.25^ns^	18.01^ns^	54.59^ns^	1.33^ns^	2.99^ns^
AR	3.11^ns^	3.13^ns^	3.07^ns^	39.85^ns^	4.33^ns^	0.22^ns^	31.84^b^	83.75^a, b^	0.17^ns^	5.64^ns^	18.72^ns^	63.29^ns^	1.38^ns^	2.77^ns^
AS1	2.97^ns^	3.26^ns^	3.15^ns^	41.59^ns^	3.58^ns^	0.22^ns^	32.47^b^	88.72^b, c^	0.16^ns^	5.38^ns^	18.76^ns^	59.24^ns^	1.20^ns^	2.23^ns^
AS2	2.95^ns^	3.34^ns^	3.25^ns^	39.94^ns^	5.33^ns^	0.21^ns^	32.20^b^	92.83^c^	0.19^ns^	5.28^ns^	19.88^ns^	56.78^ns^	1.52^ns^	2.44^ns^
BF	2.96^ns^	3.16^ns^	3.06^a^	39.59^ns^	5.96^b^	0.19^a^	30.85^ns^	85.95^ns^	0.18^b^	5.60^ns^	19.07^ns^	61.28^ns^	1.25^ns^	2.88^ns^
BF⊖	2.93^ns^	3.27^ns^	3.30^b^	39.37^ns^	3.57^a^	0.24^b^	31.40^ns^	87.22^ns^	0.16^a^	5.18^ns^	18.62^ns^	55.67^ns^	1.46^ns^	2.33^ns^
2018	3.27^b^	3.40^b^	3.50^b^	49.71^b^	7.62^b^	0.19^a^	31.22^ns^	90.35^b^	0.15^a^	6.37^b^	23.80^b^	70.90^b^	1.37^ns^	3.87^b^
2020	2.62^a^	3.03^a^	2.85^a^	29.25^a^	1.90^a^	0.24^b^	31.03^ns^	82.82^a^	0.19^b^	4.41^a^	13.88^a^	46.06^a^	1.34^ns^	1.34^a^
***p-*** **value**														
SP	0.137	0.181	0.651	0.718	0.469	0.980	0.000	0.000	0.084	0.813	0.853	0.451	0.250	0.553
BF	0.827	0.190	0.025	0.944	0.025	0.011	0.429	0.559	0.024	0.176	0.764	0.156	0.061	0.167
Y	0.000	0.000	0.000	0.000	0.000	0.016	0.783	0.000	0.000	0.000	0.000	0.000	0.801	0.000
SP × BF	0.420	0.237	0.474	0.988	0.312	0.430	0.000	0.000	0.006	0.884	0.997	0.685	0.087	0.740
SP × Y	0.000	0.000	0.000	0.000	0.000	0.078	0.000	0.000	0.000	0.000	0.000	0.000	0.489	0.000
BF × Y	0.000	0.000	0.000	0.000	0.000	0.000	0.426	0.000	0.000	0.000	0.000	0.000	0.000	0.000
SP × BF × Y	0.000	0.000	0.000	0.000	0.000	0.000	0.000	0.000	0.000	0.000	0.000	0.000	0.000	0.000

S1, sole crop of soybean; AR, alternating rows of soybean and millet; AS1, alternating strips of two rows of soybean and two rows of millet; AS2, alternating strips of two rows of soybean and four rows of millet; BF, bio-fertilizer; BF⊖, without bio-fertilizer. Different letters indicate significant difference at *p* < 0.05, ns, not significant.

Greater variations in the concentrations of macro- and micro-elements among applied treatments were present in the millet grain (Table 7). The sowing pattern significantly affected Fe accumulation, with the highest concentration noticed in AS1 (36.97 mg kg^−1^). Significant impact was expressed on Al, Cr, and Mn concentrations, too, having the highest Al and Cr values (14.09 and 0.15 mg kg^−1^, respectively) in S2, while Mn concentration was the highest in AR, with 13.59 mg kg^−1^. BF significantly increased the concentration of Ca, B, Fe, Co, Zn, and Mo (for 0.01 g kg^−1^, 1.45, 4.01, 0.01, 1.36, and 0.04 mg kg^−1^, respectively). Y, as well as SP × Y, BF × Y, and SP × BF × Y, had a significant influence on variability in the concentration of all examined elements in millet grain, while SP × BF on some of them. In regard to other elements, Fe is the only element whose concentration significantly varied by all tested factors and their interactions.

**Table 7:** Analysis of variance for the effects of sowing pattern (SP), bio-fertilizer (BF), year (Y), and their interaction on the variation of macro- and micro-elements in common millet grain.

**Sources of variation**	**Ca**	**Mg**	**S**	**B**	**Al**	**Cr**	**Mn**	**Fe**	**Co**	**Ni**	**Cu**	**Zn**	**Se**	**Mo**
	**(g kg** ^−1^ **)**			**(mg kg** ^−1^ **)**										
S2	0.12^ns^	1.46^ns^	1.35^ns^	3.18^ns^	14.09^c^	0.15^b^	12.19^a^	32.01^a^	0.05^ns^	1.59^ns^	7.14^ns^	32.24^ns^	1.75^ns^	0.17^ns^
AR	0.13^ns^	1.53^ns^	1.39^ns^	3.45^ns^	9.95^b^	0.10^a^	13.59^b^	36.94^b^	0.05^ns^	1.79^ns^	7.60^ns^	30.94^ns^	1.99^ns^	0.13^ns^
AS1	0.13^ns^	1.46^ns^	1.37^ns^	2.75^ns^	7.69^a^	0.14^a, b^	12.38^a^	36.97^b^	0.04^ns^	1.64^ns^	7.07^ns^	31.23^ns^	1.90^ns^	0.13^ns^
AS2	0.12^ns^	1.46^ns^	1.37^ns^	2.24^ns^	9.26^a, b^	0.11^a, b^	12.94^a, b^	35.26^a, b^	0.05^ns^	1.64^ns^	7.23^ns^	29.94^ns^	2.20^ns^	0.13^ns^
BF	0.13^b^	1.48^ns^	1.38^ns^	3.63^b^	10.92^ns^	0.12^ns^	12.96^ns^	37.30^b^	0.05^b^	1.71^ns^	7.33^ns^	31.77^b^	1.91^ns^	0.16^b^
BF⊖	0.12^a^	1.47^ns^	1.36^ns^	2.18^a^	9.57^ns^	0.13^ns^	12.59^ns^	33.29^a^	0.04^a^	1.62^ns^	7.19^ns^	30.41^a^	2.01^ns^	0.12^a^
2018	0.12^a^	1.64^b^	1.44^b^	3.72^b^	11.48^b^	0.09^a^	12.32^a^	33.40^a^	0.04^a^	1.46^a^	7.88^b^	32.62^b^	2.36^b^	0.19^b^
2020	0.13^b^	1.31^a^	1.29^a^	2.09^a^	9.01^a^	0.16^b^	13.23^b^	37.19^b^	0.05^b^	1.87^b^	6.64^a^	29.56^a^	1.56^a^	0.09^a^
***p-*** **value**														
SP	0.486	0.738	0.725	0.130	0.000	0.044	0.000	0.027	0.070	0.217	0.314	0.073	0.228	0.211
BF	0.000	0.941	0.406	0.000	0.143	0.317	0.185	0.002	0.014	0.206	0.551	0.028	0.554	0.040
Y	0.000	0.000	0.000	0.000	0.006	0.000	0.001	0.004	0.000	0.000	0.000	0.000	0.000	0.000
SP × BF	0.001	0.990	0.934	0.001	0.000	0.231	0.001	0.004	0.013	0.509	0.537	0.027	0.510	0.089
SP × Y	0.000	0.000	0.000	0.000	0.000	0.000	0.000	0.002	0.000	0.000	0.000	0.000	0.000	0.000
BF × Y	0.000	0.000	0.000	0.000	0.005	0.000	0.000	0.000	0.000	0.000	0.000	0.000	0.000	0.000
SP × BF × Y	0.000	0.000	0.000	0.000	0.000	0.000	0.000	0.000	0.000	0.000	0.000	0.000	0.000	0.000

S2, sole crop of millet; AR, alternating rows of soybean and millet; AS1, alternating strips of two rows of soybean and two rows of millet; AS2, alternating strips of two rows of soybean and four rows of millet; BF, bio-fertilizer; BF⊖, without bio-fertilizer. Different letters indicate significant differences at *p* < 0.05, ns, not significant.

### 3.4 Elements LER

The elements LER of intercropped soybean and common millet are shown in Table 8. In the combination of AS1 + BF, E-LER values were ≥1.5 for most elements, and only Al-LER and Mo-LER had distinctly lower values (1.10 and 1.01, respectively). In the same combination, Mn-LER was the highest (1.67). This combination provided the highest E-LER values for all elements and, in most cases, significantly higher than the other five combinations. AR + BF and AS2 + BF had LER values ≥1 for a majority of macro- and micro-elements, with exceptions of Al-LER in AR + BF and Cr-LER and Mo-LER in both variants. However, in AR + BF⊖ and AS2 + BF⊖, all E-LER values were close to 1 and, in most cases, significantly lower compared to other combinations.

**Table 8:** Elements land equivalent ratio (E-LER) depending on the different intercrop combinations and bio-fertilizer (2-year average ± standard deviation).

**E-LER**	**BF**			**BF**⊖		
	**AR**	**AS1**	**AS2**	**AR**	**AS1**	**AS2**
Ca-LER	1.13 ± 0.03^b^	1.60 ± 0.11^d^	1.14 ± 0.08^b^	1.06 ± 0.08^a^	1.27 ± 0.07^c^	1.01 ± 0.04^a^
Mg-LER	1.02 ± 0.06^a^	1.46 ± 0.13^d^	1.16 ± 0.12^b^	1.02 ± 0.02^a^	1.30 ± 0.13^c^	1.01 ± 0.04^a^
S-LER	1.00 ± 0.01^a^	1.44 ± 0.06^d^	1.10 ± 0.01^b^	0.97 ± 0.04^a^	1.20 ± 0.03^c^	0.99 ± 0.05^a^
B-LER	1.09 ± 0.09^b, c^	1.53 ± 0.11^e^	1.03 ± 0.07^b^	1.16 ± 0.13^c^	1.38 ± 0.09^d^	0.91 ± 0.11^a^
Al-LER	0.76 ± 0.16^ns^	1.10 ± 0.56^ns^	1.07 ± 0.47^ns^	0.84 ± 0.10^ns^	0.96 ± 0.29^ns^	0.98 ± 0.21^ns^
Cr-LER	0.98 ± 0.21^a^	1.50 ± 0.31^c^	0.96 ± 0.15^a^	0.93 ± 0.16^a^	1.30 ± 0.07^b^	0.82 ± 0.07^a^
Mn-LER	1.19 ± 0.06^b^	1.67 ± 0.07^d^	1.28 ± 0.11^c^	1.09 ± 0.05^a^	1.34 ± 0.06^c^	1.05 ± 0.04^a^
Fe-LER	1.11 ± 0.04^a^	1.66 ± 0.03^d^	1.28 ± 0.08^b^	1.07 ± 0.05^a^	1.34 ± 0.08^c^	1.07 ± 0.03^a^
Co-LER	1.14 ± 0.08^b^	1.50 ± 0.09^d^	1.32 ± 0.13^c^	0.93 ± 0.02^a^	1.07 ± 0.17^b^	0.92 ± 0.06^a^
Ni-LER	1.10 ± 0.04^b^	1.46 ± 0.06^d^	1.14 ± 0.04^b^	1.10 ± 0.09^b^	1.28 ± 0.07^c^	0.98 ± 0.07^a^
Cu-LER	1.04 ± 0.01^a^	1.47 ± 0.07^d^	1.18 ± 0.08^b^	1.06 ± 0.05^a^	1.29 ± 0.11^c^	1.06 ± 0.02^a^
Zn-LER	1.08 ± 0.06^b^	1.55 ± 0.11^d^	1.07 ± 0.02^b^	1.10 ± 0.08^b^	1.26 ± 0.02^c^	0.98 ± 0.08^a^
Se-LER	1.07 ± 0.26^a^	1.53 ± 0.12^c^	1.16 ± 0.21^a, b^	1.17 ± 0.18^a, b^	1.19 ± 0.11^a, b^	1.33 ± 0.30^b, c^
Mo-LER	0.82 ± 0.02^a^	1.01 ± 0.02^c^	0.81 ± 0.09^a^	0.95 ± 0.13^b, c^	0.99 ± 0.04^c^	0.87 ± 0.13^a, b^

AR, alternating rows of soybean and millet; AS1, alternating strips of two rows of soybean and two rows of millet; AS2, alternating strips of two rows of soybean and four rows of millet; BF, bio-fertilizer; BF⊖, without bio-fertilizer. Different letters indicate significant difference at *p* < 0.05, ns, not significant.

### 3.5 PCA

**Figure 1:**
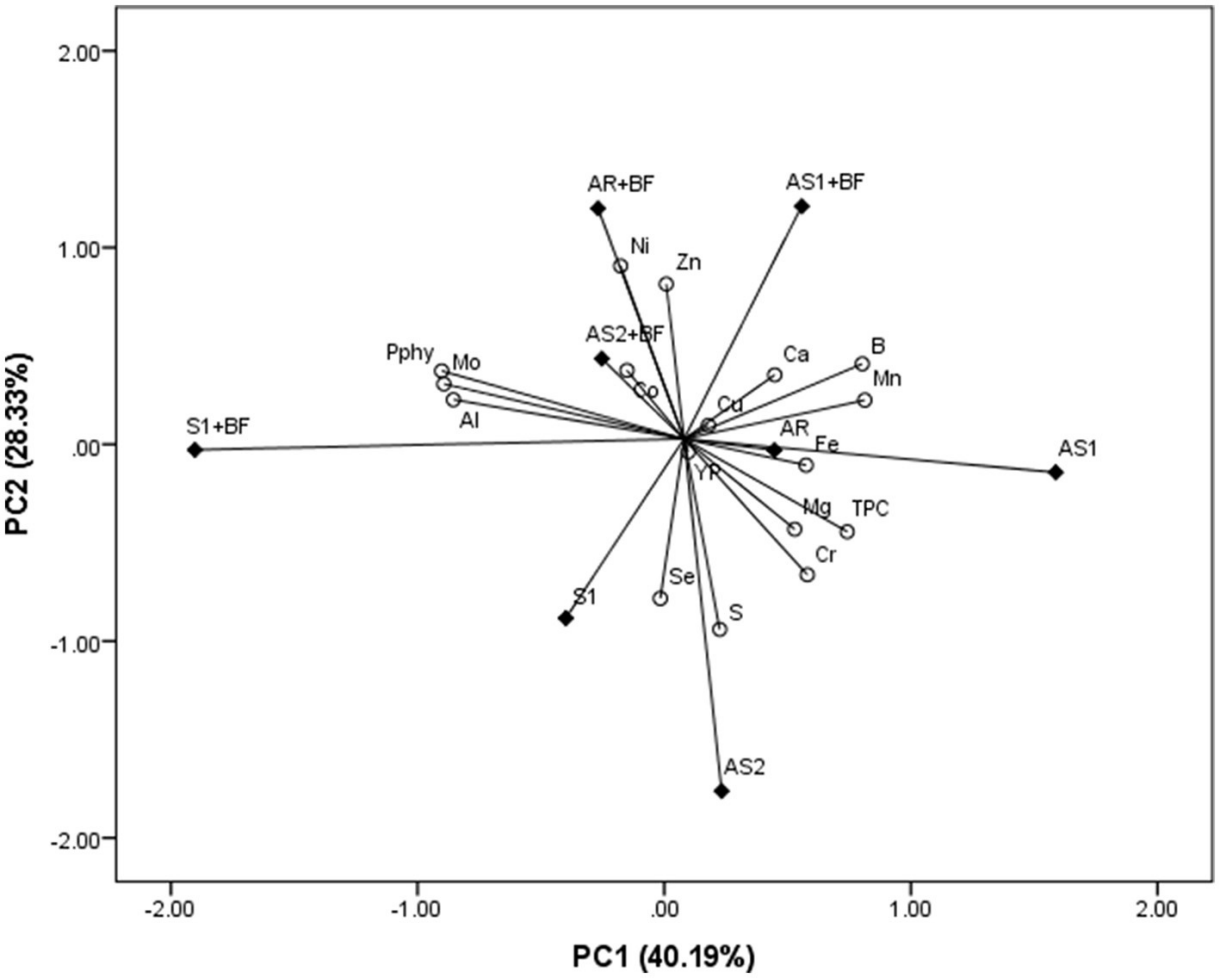
Principal component analysis for phytate phosphorus (Pphy), total phenolic compounds (TPC), yellow pigment (YP), and macro- (Ca, Mg, and S) and micro-elements (B, Al, Cr, Mn, Fe, Co, Ni, Cu, Zn, Se, and Mo) concentration in soybean influenced by different intercrop combinations (AR, alternating rows of soybean and millet; AS1, alternating strips of two rows of soybean and two rows of millet; AS2, alternating strips of two rows of soybean and four rows of millet), as well as sole crop (S1) and bio-fertilizer (BF).

To evaluate the joint impact of SP and BF on Pphy, TPC, YP, as well as macro- and micro-element concentrations, PCA was applied. For soybeans, it resulted in the four-component model, which explained 93.50% of the overall variability. PC1 and PC2 explained 40.19 and 28.33% of total variability, respectively, and their score and loadings plot are mutually shown in Figure 1. TPC, B, Mn, Fe, Cr, Mg, and Ca correlated positively with PC1, while Pphy, Mo, and Al correlated negatively. The positive correlation with PC2 had Ni and Zn, while S and Se correlated negatively. The concentration of Ni and Zn in soybean grain corresponded greatly with the AR + BF combination, while Co accumulation in grain was mostly affected by the AS2 + BF combination. Ca concentration was influenced by AS1 + BF, and accumulation of Al, Mo, and Pphy by the S1 + BF combination. Considering BFØ treatments, AR and AS1 controlled Fe accumulation and, to a lesser extent, accumulation of Mn, Mg, Cr, B, and TPC. S and Se concentrations were separated by the AS2, while only Se was influenced by S1, but to a lesser degree. Taking into account the mutual projection of PC1 and PC2, two groups of samples could be observed. One group consists of combinations with BF, while the second group has combinations without BF. In the first group, intercropping is separated from the sole crop and forms a subgroup, while samples in the second cluster are dissipated, with similarity shown only between AR and AS1.

**Figure 2:**
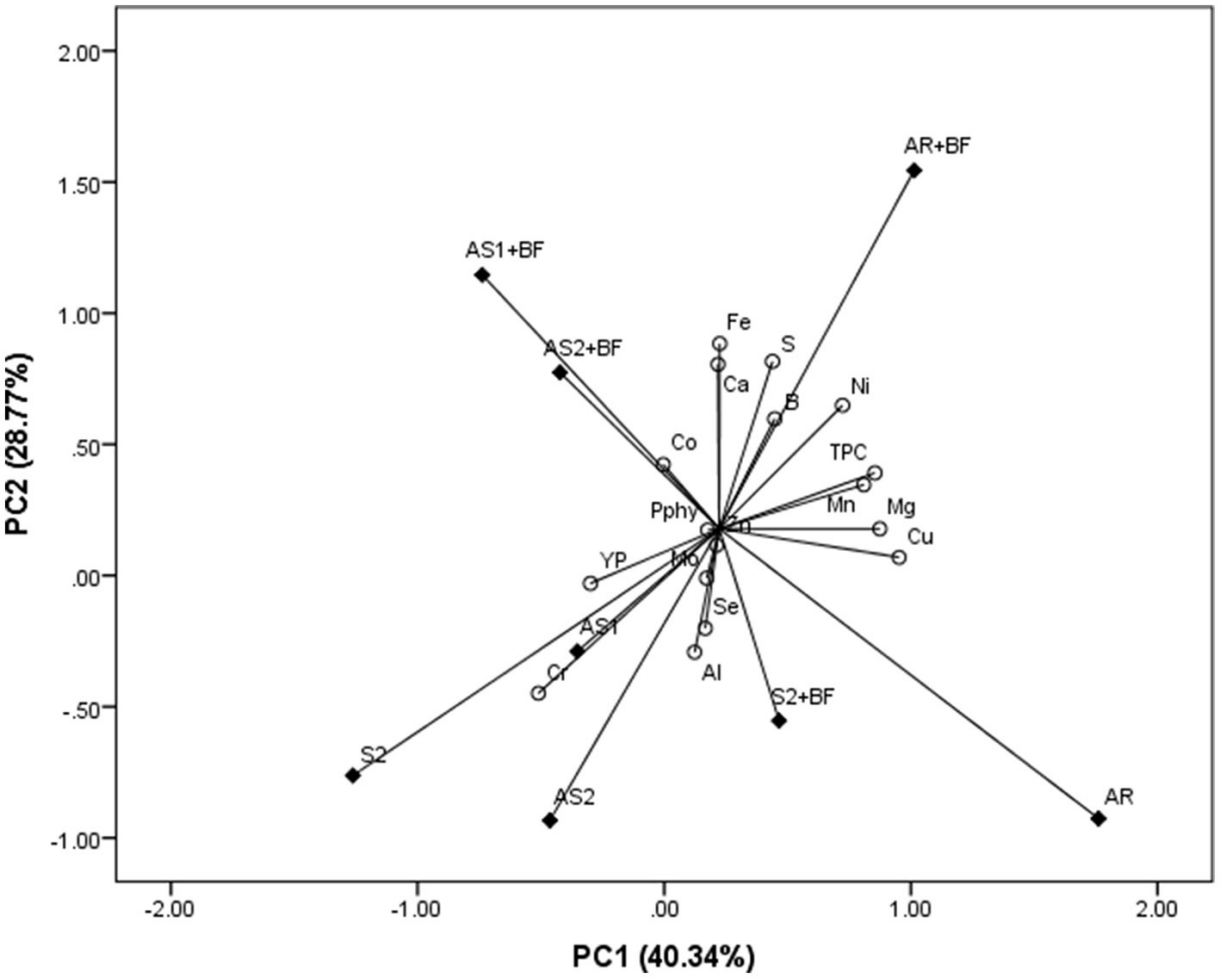
Principal component analysis for phytate phosphorus (Pphy), total phenolic compounds (TPC), yellow pigment (YP), and macro- (Ca, Mg, and S) and micro-elements (B, Al, Cr, Mn, Fe, Co, Ni, Cu, Zn, Se, and Mo) concentration in millet influenced by different intercrop combinations (AR, alternating rows of soybean and millet; AS1, alternating strips of two rows of soybean and two rows of millet; AS2, alternating strips of two rows of soybean and 4 rows of millet), as well as sole crop (S2) and bio-fertilizer (BF).

PCA revealed the four-component model for common millet, which explained the 90.40% of total variability, whereas PC1 and PC2 were described as 40.34 and 28.77% of total variability, respectively. The results presented in Figure 2 showed that Cu, Mg, Mn, Ni, and TPC positively correlated with PC1, while Ca, S, B, Ni, and Fe in the same direction correlated with PC2. AS1 + BF and AS2 + BF combinations were determined by Co accumulation, while AR + BF affected B accumulation in grain and, to a lesser extent, S and Ni concentrations. Al, Se, and Mo concentrations were under an impact of the S2 + BF combination. Cr and YP accumulation were affected by AS1 and slightly by S2 combinations. AS2 influenced the accumulation of Al, Se, Mo, and Cr in millet grain, but to a lesser extent. Regarding clustering, two groups are also formed in the case of millet. One cluster consists of intercrop combinations integrated with BF, while the second cluster includes S2, AS1, and AS2 combinations. S2 + BF and AR are lying outside these two clusters, which is why they are considered outliers. In the first group, the similarity between AS1 + BF and AS2 + BF is observed, and these two combinations consist of one subgroup.

### 3.6 Molar ratios between phytic acid and essential elements

**Figure 3:**
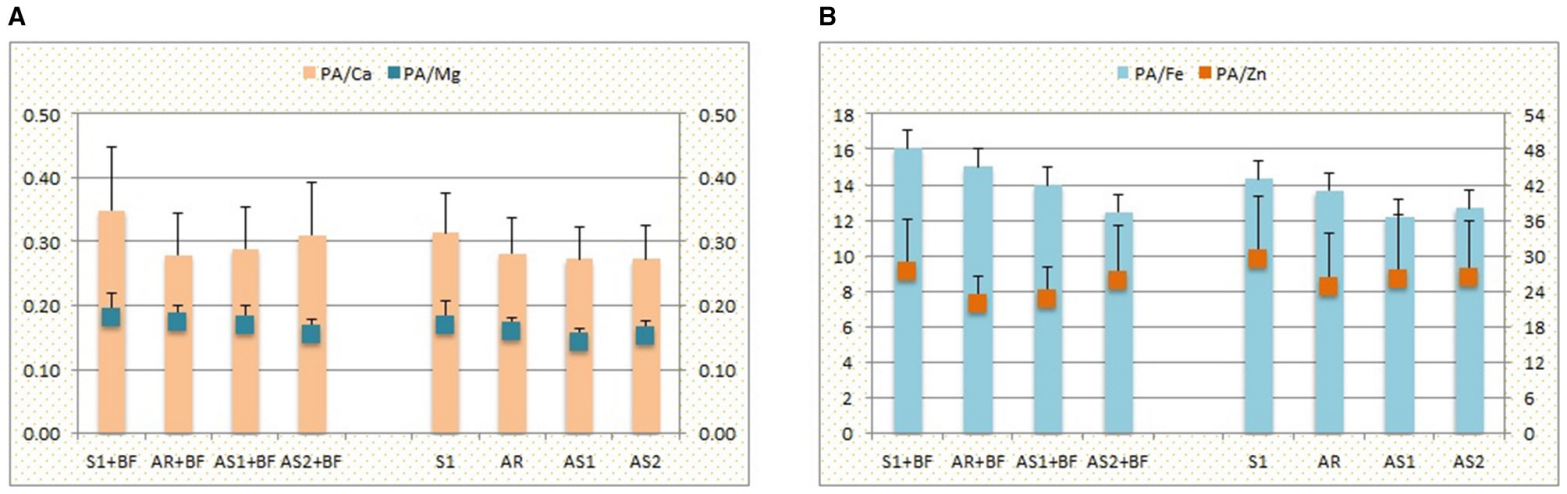
The effect of sowing pattern (S1, sole crop; AR, alternating rows of soybean and millet; AS1, alternating strips of two rows of soybean and two rows of millet; AS2, alternating strips of two rows of soybean and four rows of millet) in combination with bio-fertilizer (BF) on the molar ratios between phytic acid (PA) and **(A)** Ca and Mg, **(B)** Fe and Zn, for soybean (2-year average + standard deviation).

The intercropping and BF affected molar ratios between phytic acid and essential elements, such as Ca, Mg, Fe, and Zn. Regarding soybean (Figure 3), all ratios had lower values in intercrops comparing to the sole crop, irrespective of whether BF was applied or not (having the highest values of 0.35, 0.18, 16.07, and 27.44 for PA/Ca, PA/Mg, PA/Fe, and PA/Zn, respectively, in S1 + BF; and 0.31, 0.17, 14.35, and 29.37 in S1). The lowest values were obtained for PA/Ca in AS1 and AS2 (0.27) and for PA/Mg and PA/Fe in AS1 (0.14 and 12.19, respectively), while the lowest PA/Zn ratio (21.94) was in AR + BF.

**Figure 4:**
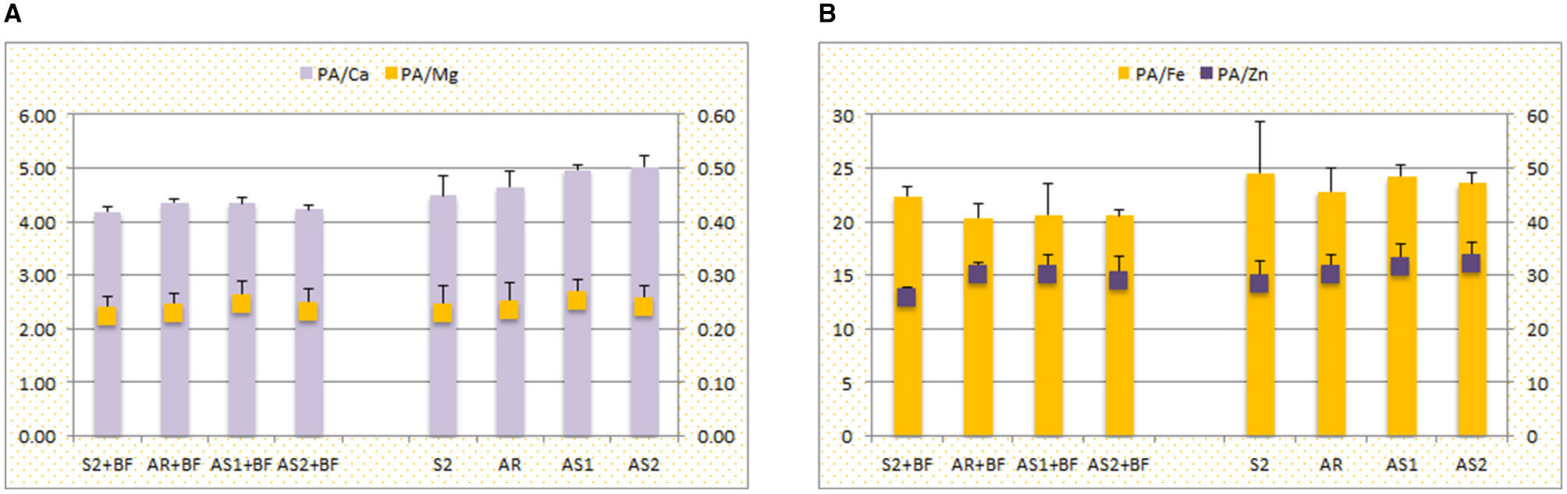
The effect of sowing pattern (S2, sole crop; AR, alternating rows of soybean and millet; AS1, alternating strips of two rows of soybean and two rows of millet; AS2, alternating strips of two rows of soybean and four rows of millet) in combination with bio-fertilizer (BF) on the molar ratios between phytic acid (PA) and **(A)** Ca and Mg, **(B)** Fe and Zn, for common millet (2-year average + standard deviation).

Opposite to soybean, common millet grain ratios of PA/Ca, PA/Mg, and PA/Zn had the lowest values in S2 + BF (4.18, 0.22, and 25.81, respectively), while PA/Fe ratio was the smallest in AR + BF (20.37) (Figure 4). Generally, the smaller values of ratios were obtained in intercrops with BF, except PA/Mg ratio, where variations were negligible.

## 4 Discussion

### 4.1 Meteorological factors as a source of variability

With the aim to develop successful production technology, resilient to climate change, this research was performed under dry land conditions, investigating whether the intercropping and BF are good strategies to mitigate meteorological variations and support food security. Together with the fact that millet is a resilient crop (22) with water use efficiency higher than other C4 crops (37), a significantly higher GY was achieved in 2018, with a lesser impact on soybean GY. Explanation could be found in higher temperatures in the heading stage of development and higher precipitation during the grain-filling period, as both factors have an important role in achieving higher yields of millet grain (38). However, as an insignificant influence of year on LER values is observed, the importance of this research was reflected through the potential yield stability of soybean–common millet intercropping in different climatic conditions.

Variability in grain components influenced by year was significant for both crops. The low precipitation level in July 2020 could be a reason for the greater accumulation of Pphy, TPC, and YP, as antioxidants in the grain of both crops, since variations in their concentrations are distinctly dependable to the environmental conditions (39–41). Thus, this research proved that reduced yield potential was compensated with a greater level of all antioxidants as well as some of the elements in grains of both soybean and millet, similar to the previously obtained results by Dragicevic et al. (42) and Bartwal et al. (43).

### 4.2 Intercropping as a source of variability

Intercropping of millet and legumes has numerous benefits regarding crop productivity and resource use efficiency, thus supporting agricultural sustainability (44). Considering a lack of data for common millet–soybean intercropping, the obtained LER values >1 for all planting patterns indicate enhanced productivity under dry land conditions and low-input systems, signifying the importance of this growing model for yield height and stability. While Ijoyah et al. (45) pointed out that intra-row spacing of pearl millet (*Pennisetum glaucum* L.) is one of the key factors that affect the productivity of intercrops, this study pointed out that SP, in general, plays an important role in the greater LER achievement. Ahmadvand and Hajinia (23, 25) also attained LER values >1 for all planting patterns and proved the beneficial effect of combining common millet and soybean in intercropping. The highest LER value achieved in AS1 underlined 1:1 ratio set as alternating strips as the most perspective to achieve higher total yield and promote land utilization compared with sole crops. What is more, the same combination contributed mostly to the increase of YP concentration in millet grain, thus increasing its quality, including a potential bio-availability of essential elements for humans (19, 20). The PCA proved that YP was affected by AS1, too. In addition to YP, Pphy and TPC, as important antioxidants, were also increased in millet grain by AS1, thus favoring it from the health point of view. However, PCA showed that the compact group (consisting of S2, AS1, and AS2 sowing patterns) was positively connected with the concentration of YP in millet grain, but this group is in the opposite direction with TPC. This can be explained by the influence of the AR sowing pattern, which is separated from this group, where millet from this combination had significantly higher values of TPC compared to the other three variants. Based on this, it can be said that in the AR combination, the influence of soybean is of the utmost importance when the quality of millet grain is considered, while in the other three combinations, the dominant influence had millet. Still, PA and TPC are anti-nutrients, reducing the potential bio-availability of essential elements, so their concentration should be balanced regarding the beneficial and adverse effects (17). Enhanced Pphy in millet in the presence of soybean confirmed that P uptake by cereals is pronounced in the presence of legumes, especially in low-input systems (46), while the greatest TPC concentration (part of the stress response) (40) in AR leads to presume that millet has been stressed by soybean competitively in this combination, which was additionally supported by the lowest GY value.

In regard to elements concentration in grains of both crops, intercropping significantly increased the accumulation of essential elements lacking in the diet, thereby contributing to the mitigation of global malnutrition. Only for Zn, intercropping was insignificant. In soybean grain, the greatest Mn and Fe accumulation was in AS1 and AS2, respectively, while in millet, the lowest Mn and Fe values were noticed in AS1 and AS2 regarding other intercrop combinations. PCA has additionally revealed the importance of concentrations of these two elements in soybean grain by AR and AS1 combinations. Therefore, irrespective of the presence of potential competitiveness, facilitation between soybean and millet regarding nutrient use efficiency was present at the same time. Furthermore, AS1 singled out this planting pattern as the most promising for nutrient yield response. Explanation could be found in the possibility of gramineous plants to overcome Fe, Mn, and Zn deficiency by exudating phytosiderophores, thus enhancing their availability to companion crops in intercrop too (47, 48). It is important to underline that there was significantly lower accumulation of Al and Cr in intercropped millet. Lower Al accumulation, particularly in AS1, where inter-row distance was the smallest, could be the result of the ability of soybean roots to excrete citrate and the ability of symbiotic rhizobia to produce siderophores, both reducing Al uptake (49, 50). This result presents novelty, giving a particular advantage of combining common millet and soybean in intercrop to raise nutritional quality and foster sustainability by reducing heavy metals load in crops.

Despite the fact that grains of cereals and legumes are, in general, rich in essential minerals, their accessibility by humans is highly dependent on the status of anti-nutrients and promoters (51, 52), which is closely related to variability in examined antioxidants, especially PA (20, 53, 54). Therefore, the molar ratio between PA and essential metals is an important indicator of their potential bio-availability (55). Maares and Haase (56) claimed that Zn bio-availability directly depends on its ratio with PA, and Gibson et al. (57) confirmed the dependence of Fe, Zn, and Ca absorption on PA levels. According to the presented results, PA/Ca, PA/Mg, PA/Fe, and PA/Zn ratios were lower in intercropped soybean than in the sole crop, supporting the ability of roots of gramineous crops to promote mineral availability by exudates (42, 58). Although the concentration of Pphy and elements in soybean grain were not significantly affected by intercropping (except Fe concentration), the values of ratios implied that intercropping could be the way to improve desirable nutritive traits of soybean grain. In contrast, lower values of PA/Ca, PA/Mg, and PA/Zn ratios in sole millet revealed it as a better source of available essential elements in the diet, but still not satisfactory as these ratios are quite high compared to the desirable ones (59). However, the smaller value of PA/Fe ratio in intercropped millet in combination with the highest YP concentration in AS1 may additionally promote Fe bio-availability, as the food matrix overall is responsible for element accessibility by the human organism (20), but detailed investigations are needed to confirm it.

### 4.3 Bio-fertilizer as a source of variability

Excessive use of chemical fertilizers in large-scale production has led to environmental pollution, simultaneously causing numerous health hazards. Therefore, an ecological solution was required, such as bio-fertilizers, which could be successfully used to increase the absorption and accumulation of important nutrients in edible parts of plants, thereby supporting high yields and nutrient density in crops in a sustainable way (60, 61). A lot of studies have examined the impact of bio-fertilizers on soybean–cereals, as well as millet–legume intercropping, with variable results (42, 62, 63), but research on common millet and soybean intercropping was not published yet. Therefore, this study showed for the first time results from field trials of common millet–soybean intercropping in combination with BF.

Results showed negligible effect of BF solely on GY and LER value, but mutual interaction of intercropping and BF significantly affected both. Considering nutritional quality, this interaction was more significant for millet, which is proven with PCA, too. All intercrop combinations with BF were separated in one group, with high similarity between AS1 + BF and AS2 + BF regarding AR + BF. Integrated effects of AR and BF were the most liable for TPC variation in millet grain, emphasizing the increased impact of soybean in the presence of BF. In relation to the concentration of antioxidants, BF alone expressed a significant impact only on Pphy concentration in soybean grain, increasing the average value to 0.21 mg g^−1^. This is not surprising due to the fact that PGPR, AMF, and *Trichoderma* spp. are beneficial for P availability in the soil and consequently its absorption by the crops (64–66). In the same manner, *Azotobacter* strains are able to increase P accumulation in soybean grains (67). PCA for soybean proved this fact, as all combinations with BF were separated in one group. However, a subgroup in this group is also observed, where intercrops integrated with BF were distinguished from S1 + BF, which confirms that millet had an influence on examined parameters in soybean grain, too. Based on the above, it can be concluded that the dominant effect of soybean on the quality of millet grain, as well as the effect of millet on the quality of soybean grain, was pronounced in the presence of BF.

The beneficial effect of BF on the potential bio-availability of minerals in grains was supported by smaller values of ratios between PA and essential elements achieved in millet grains. On the other hand, the higher values of PA/Ca, PA/Mg, and PA/Fe in soybeans could be attributed to the greater P uptake through BF application, as discussed above. Although BF increased Pphy in soybean grain and thus the quality regarding availability of essential elements was reduced, its role as an antioxidant should be taken into account, contributing to the overall antioxidant potential of soybean. Nevertheless, lower values of PA/Zn ratio in soybean subjected to BF were a consequence of higher, but not significantly, Zn concentration in grain and confirm previous research conducted by Mostafavian et al. (68), who showed insignificant influence of BF on Zn variability. Thus, this research pointed out the cumulative effect of matrix components on the increased potential of Zn bio-availability.

A significant impact of BF was noticed for element concentration in grains of both crops. In soybean, Al and Co concentrations increased, while S and Cr concentrations decreased by BF application, while millet accumulated greater concentrations of Ca, B, Fe, Co, Zn, and Mo in treatment with BF. Based on this and the fact that highly dependent plants show a strong, and less dependent plants show a weak, nutrient response to AMF inoculation (69), it could be supposed that the common millet is the high-dependent (considering that 6 out of 14 examined elements are dependent on microbial inoculation), while soybean is the less-dependent plant to microbial inoculation. Consequently, this type of BF has the potential to be used for millet biofortification, boosting the accumulation of important micro-elements in grain. Additionally, it should be emphasized that the mutual interaction of intercropping and BF had a significant impact on the accumulation of Fe and Mn in soybean and Fe, Mn, and Zn in millet grains. Due to the general scarcity of these elements in calcareous soils (47), deficiency regarding these elements (70) could be exceeded. As AR + BF mostly affected Zn variability in soybean grain and Fe and Mn variability in millet grain, these findings highlight the importance of combining intercropping and BF to manage the nutritional quality of grains, pointing out 1:1 ratio as the most advantageous for soybean and millet. Dragicevic et al. (42) also revealed significant variations of Fe and Zn concentrations in grains of intercropped maize and soybean, particularly when bio-fertilizer was applied, emphasizing the potential of integrated practice as a promising way of biofortification. Nevertheless, BF-promoting effect should be studied further, including its effect on the soil quality and changes in microbial communities, not just grain quality, which is the foreground of this study.

Regarding E-LER, the results additionally supported the positive effect of intercropping + BF to improve the efficiency of nutrient usage from the soil. For all examined elements, the highest E-LER values were obtained by AS1 + BF, giving the advantage to this combination over others. This combination also contributed to the greater efficiency of Al, Co, and Ni, making them less desirable. Irrespective of the fact that E-LER points their greater accumulation in intercrops + BF, it must be taken into account that heavy metals toxicity is related to their cumulative effect from different food sources and that in trace amounts, they are essential for both plants and humans (71, 72). Alizadeh et al. (73) recommended the same planting pattern for linseed–faba bean intercropping, especially in combination with bio-fertilizer, to increase nutrient-LER and improve the biological characteristics of the soil. Similar results were obtained for intercropped fenugreek and buckwheat, where the total uptake of N and P per land area was greater in intercropping, highlighting a 2:1 ratio (F:B) as the favorable (74). The results of this study confirmed that the greatest effectiveness for crop performance boosting revealed the interaction of the sowing pattern (intercropping) and the use of a bio-fertilizer.

## 5 Conclusion

This research present novelty as common millet–soybean intercropping integrated with the bio-fertilizer (without any other inputs), has not been investigated yet, and information about effects of applied practices on yield and quality parameters are presented for the first time.

According to the LER values, a 1:1 ratio set as alternating strips could be the most prominent combination to increase yield under dry land conditions and low-input systems. The same planting pattern raised nutrient yield response too, while the implementation of bio-fertilizer additionally boosted nutrients outtake with grain yield. In addition, a higher accumulation of essential micro-elements, such as Fe, Zn, B, Co, and Mo, in millet grain was supported by greater potential bio-availability of important elements (Ca, Fe, and Zn) with bio-fertilizer application. On the other hand, intercropping influenced higher potential bio-availability of Ca, Mg, Fe, and Zn in soybean grain, simultaneously enhancing its quality. Concerning the integrated effect of intercropping and bio-fertilizer, the potential for enhanced use of agro-ecosystem services was revealed. This could be promising in terms of biofortification due to the significant impact of the applied combination on concentration of elements, which mainly lacked in human diet, including their potential bio-availability.

The novelty and contribution of this study were revealed through the practical solution toward improved land utilization regarding yield and elements accumulation driven by soybean–common millet intercropping integrated with bio-fertilizer, simultaneously enhancing the nutritional value of both crops under low-input and dry land conditions, supporting sustainability, at the same time. Thus, other crops could also be included in intercropping experiments, as well as various microbial consortia, as potential bio-fertilizers.

## Data availability statement

The original contributions presented in the study are included in the article/supplementary material, further inquiries can be directed to the corresponding author.

## Author contributions

MŠ: Data curation, Investigation, Methodology, Writing—original draft, Formal analysis, Software. MS: Conceptualization, Funding acquisition, Project administration, Resources, Supervision, Visualization, Writing—review & editing. DM-O: Conceptualization, Resources, Supervision, Visualization, Writing—review & editing, Methodology. MB: Methodology, Writing—review & editing, Data curation, Investigation, Software. MT: Writing—review & editing, Funding acquisition, Resources. IK: Writing—review & editing, Data curation, Formal analysis, Investigation, Methodology, Validation. VD: Data curation, Investigation, Methodology, Conceptualization, Supervision, Visualization, Writing—original draft.
